# Resection vs. Sorafenib for Hepatocellular Carcinoma With Macroscopic Vascular Invasion: A Real World, Propensity Score Matched Analytic Study

**DOI:** 10.3389/fonc.2020.00573

**Published:** 2020-05-05

**Authors:** Jie Mei, Shao-Hua Li, Qiao-Xuan Wang, Liang-He Lu, Yi-Hong Ling, Jing-Wen Zou, Wen-Ping Lin, Yu-Hua Wen, Wei Wei, Rong-Ping Guo

**Affiliations:** ^1^Department of Hepatobiliary Oncology, Sun Yat-sen University Cancer Center, Guangzhou, China; ^2^State Key Laboratory of Oncology in South China, Guangzhou, China; ^3^Collaborative Innovation Center for Cancer Medicine, Guangzhou, China; ^4^Department of Radiation Oncology, Sun Yat-sen University Cancer Center, Guangzhou, China; ^5^Department of Pathology, Sun Yat-sen University Cancer Center, Guangzhou, China

**Keywords:** hepatocellular carcinoma, macroscopic vascular invasion, resection, sorafenib, propensity score matched

## Abstract

**Background:** Macroscopic vascular invasion (MVI) commonly occurs in patients with advanced hepatocellular carcinoma (HCC) for which resection and sorafenib are the common therapies prescribed. Here, we aimed to compare the survival outcomes of these two therapies in HCC patients with MVI.

**Methods:** In total, 496 patients diagnosed with HCC and MVI without extrahepatic metastasis, treated with resection (resection-based group, *n* = 388) and sorafenib (sorafenib-based group, *n* = 108) were included in this study. A one-to-one propensity score-matching analysis (PSM) was performed to minimize the effect of potential confounders.

**Results:** The median OS in the resection- and sorafenib-based group was 20.7 months (95% CI: 16.9–24.5) and 11.6 months (95% CI: 8.4–14.9) (*p* < 0.001), respectively. The median PFS was 4.7 months (95% CI: 3.8–5.5) in the resection-based group and 4.4 months (95% CI: 3.6–5.2) in the sorafenib-based group (*p* < 0.001). After PSM, 72 patients from each group were matched. The median OS was 27.2 months (95% CI: 16.4–38.0) in the resection-based group and 13.0 months (95% CI: 9.6–16.3) in the sorafenib-based group (*p* < 0.001). The median PFS was 5.3 months (95% CI: 3.2–7.4) in the resection-based group and 4.8 months (95% CI: 3.6–6.0) in the sorafenib-based group (*p* = 0.061).

**Conclusion:** Findings from this study showed that, compared with sorafenib-based treatment, surgical resection might be associated with better survival benefits to HCC patients with MVI.

## Introduction

Hepatocellular carcinoma (HCC) is one of the most common malignancies, and the fourth leading cause of cancer-related deaths worldwide (1). Because of its concealed onset, HCC often progresses to macroscopic vascular invasion (MVI) at the time of diagnosis (2). Before new targeted drugs such as lenvatinib and immune checkpoint inhibitors were available, the first-line of treatment for HCC patients with MVI recommended by the Barcelona guideline was systemic therapy with sorafenib (3) and had a median overall survival (mOS) ranging from 5.6 to 8.1 months (4, 5). However, in the Asia-Pacific region, some patients with MVI, especially those without extrahepatic metastases, could still benefit from survival through resection, with mOS ranging from 8.9 to 33 months (6–11). Therefore, the optimal choice between the two therapies for HCC patients with MVI was controversal.

To date, only a few studies have investigated the prognosis of surgical resection in comparison to sorafenib in HCC patients with MVI and inconsistent results have been reported, possibly, due to imbalanced patients characteristics between the investigated cohorts and a limited number of enrolled patients (8, 12). Nowadays, the vast majority of patients with MVI were not in the initial treatment state when receiving surgery or sorafenib. Late-stage HCC patients are referred to combined therapies, instead of surgery or sorafenib alone. Therefore, relevant clinical researches were valuable.

The purpose of this study was to compare the prognoses of HCC patients with MVI undergoing surgical resection and sorafenib, aiming to provide a reference for the treatment of advanced HCC patients.

## Materials and Methods

This study was conducted according to the ethical guidelines of the 1975 Declaration of Helsinki. The analysis of the patient data was reviewed and approved by the Institutional Review Board and Human Ethics Committee at the Sun Yat-sen University Cancer Center (SYSUCC; Guangzhou, China).

### Patients

**Figure 1:**
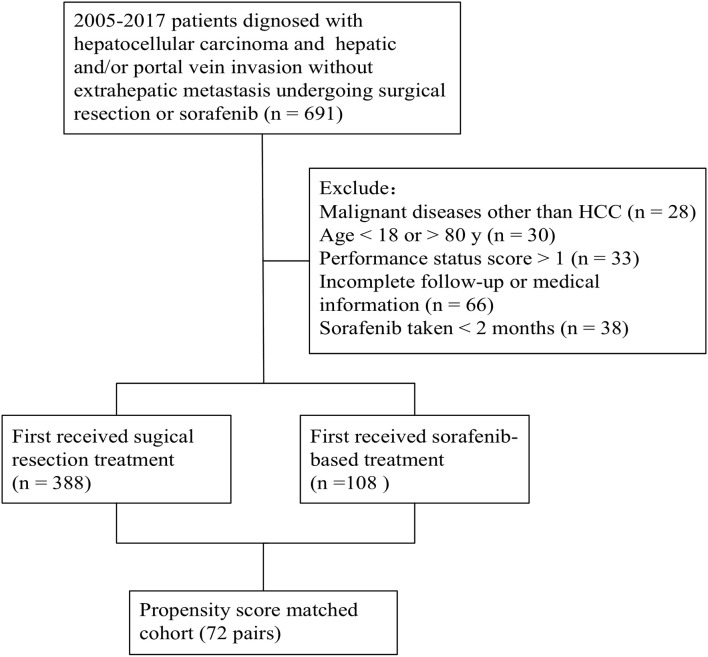
Patient enrolment and categorization flow chart.

The medical records of patients diagnosed with HCC and MVI without extrahepatic metastasis who underwent surgical resection or were prescribed sorafenib as part of standard therapy at the Department of Liver Surgery (SYSUCC), between 2005 and 2017, were reviewed for eligibility. Some patients were excluded based on the following criteria: (a) diagnosed with malignant diseases other than HCC; (b) aged >80 or <18; (c) had a performance status score >1; (d) had incomplete follow-up or medical information; (e) had sorafenib treatment for <2 months. Those who first received surgical resection treatment were classified into a resection-based group, while those who first received sorafenib-based treatment were classified into a sorafenib-based group. The patient enrolment and categorization flow chart is shown in Figure 1. All laboratory serum test data was collected within 3 days before treatment (resection or sorafenib). Preoperative imaging examinations included contrast-enhanced computed tomography (CT) or magnetic resonance imaging (MRI) within a week before treatment.

### Treatment Procedure

Hepatic resection was performed as previously described (13). Intraoperative ultrasound was routinely performed to evaluate the tumor burden, remnant liver, and possibility of a negative resection margin. Anatomic hepatectomy with mass tumor thrombectomy was the preferred method of liver resection. Depending on its location and extent, the tumor thrombus was removed by en-bloc resected with the tumor tissue or extracted from the lumen of the blood vessel. Tumor thrombus was confirmed by rinsing with normal saline, and the absence of tumor thrombus formation was confirmed by intraoperative ultrasound.

Sorafenib (Bayer, Leverkusen, Germany) was initially orally administered 200 or 400 mg twice daily and continued for at least 2 months. Withdrawal and reduction of the drug depended on unacceptable toxicities or untreatable disease progression.

The final follow-up ended on July 31, 2019. Enhanced CT or MRI was performed every 2 or 3 months after surgery or sorafenib according to subsequent therapies. Follow-ups were performed as previously described (14), unless judged otherwise by the treating physicians.

### Diagnosis and Definitions

The diagnosis of MVI was based on standard radiological imaging prior to treatment (resection or sorafenib prescription) (15, 16). Portal vein tumor thrombus (PVTT) was graded according to the classification suggested by the Liver Cancer Study Group of Japan (17). Based on liver vessel structure and prognosis for different location of vascular tumor thrombus (18), we combined hepatic vein tumor thrombus (HVTT) into PVTT classification, based on which, Vp1 represented the invasion of a third-order branch or distal to the second branch of the portal vein; Vp2, invasion of a second-order branch of the portal vein, or branch of the hepatic vein; Vp3, invasion of in the first branch of the portal vein, or hepatic vein trunk or the short hepatic vein; Vp4, invasion of the main trunk/controlateral branch of the portal vein, or inferior vena cava. Overall survival (OS) was defined as the time interval from treatment initiation to cancer-related death. Progression-free survival (PFS) was defined as the time interval from treatment initiation to tumor progression. For the resection-based group, tumor progression was defined as intrahepatic recurrence or new intrahepatic or extrahepatic lesions developed. For the sorafenib-based group, progression was defined as progressive disease (PD) according to the modified response evaluation criteria in solid tumors (mRECIST) (19), progressive intrahepatic tumor thrombus or extrahepatic metastasis.

### Statistical Analysis

Categorical variables in baseline characteristics were compared using the Pearson's χ^2^ test or Fisher's exact test. To minimize the influence of selection bias produced by preoperative factors between the two groups, propensity score matching (PSM) was conducted using a logistic regression model (20, 21). Pre-treatment variables were entered into the PSM, comprising of age (≤/>50 years old), gender (male/female), hepatitis B surface antigen DNA (HBs DNA) (≤103/>103), liver cirrhosis (None or mild/Moderate or severe), aspartate aminotransferase (AST) (≤50/>50 U/L), albumin (ALB) (≤40/>40 g/L), total bilirubin (TBIL) (≤20.5/>20.5 μmol/L), prothrombin time (PT) (≤13.5/>13.5 s), Child-Pugh score (5/>5), tumor number (1/>1), largest nodule (<5/5-10/>10 cm), distribution (Uni-lobar/Bi-lobar), tumor thrombus (Vp1/2/3/4). PSM was performed by a 1:1 matching method with a caliper width of 0.1. Survival analyses were calculated using the Kaplan-Meier method and differences in survival curves were analyzed using the log-rank test. All variables with a *P* < 0.1 in univariate analyses were used in multivariate analyses using the Cox's proportional hazards models. The hazard ratio (HR) and confidence intervals (CI) were also calculated. A value of two-tailed *P* < 0.05 was considered statistically significant. All data analyses were performed using SPSS 25.0 software (SPSS Inc., Chicago, IL) and GraphPad Prism (version 8.0; GraphPad, Inc.).

## Results

### Identification of Study Patients

From 2005 to 2017, 488 patients with HCC who underwent surgical resection (*n* = 388) or sorafenib (*n* = 108) treatment after a diagnosis of MVI without extrahepatic metastasis were identified. Of note, all patients in the sorafenib-based group were treated since January 2009 because sorafenib was available only from that year. In the resection-based group, 88 (22.7%) patients underwent surgical resection before January 2009 and the rest after January 2009.

### Characteristics of the Study Patients

Between 2005 and 2017, 691 patients were reviewed for eligibility and 496 patients were ultimately included in this study (388 in resection-based group, 108 in sorafenib-based group).

The clinical pre-treatment characteristics of the patients in the resection-based and sorafenib-based groups are summarized in Table 1. In general, patients who underwent surgical resection had smaller tumor burden and better liver function. In the resection-based group, a smaller proportion of patients had severer liver cirrhosis (51.0 vs. 78.7%, *p* < 0.001), higher child-pugh score (11.3 vs. 24.1%, *p* < 0.001), higher AST (61.1 vs. 75.0%, *p* = 0.008), and higher TBIL (13.7 vs. 28.7, *p* < 0.001), as compared to the sorafenib-based group. Meanwhile, larger proportion of patients in the sorafenib-based group were with multiple (67.6 vs. 32.2%, *p* < 0.001) or bilateral tumors (50.9 vs. 9.5%, *p* < 0.001), and had higher tumor thrombus grade (Vp3 and Vp4, 90.8 vs. 65.7%, *p* < 0.001). 313(80.7%) patients received surgical resection as their first treatment in the resection-based group, while only 40 (37.0%) patients were first treated with sorafenib in sorafenib-based treatment. In this study, 25 (6.6%) patients received subsequent sorafenib treatment in the resection-based group, while 3 (2.8%) patients chose surgical resection afterward in the sorafenib-based group.

**Table 1:** Baseline clinical characteristics of patients before PSM.

Characteristic^*^	**Resection-based (*n* = 388)**	**Sorafenib-based (*n* = 108)**	***P-*value**
Age (y)			0.064
≤50	215 (55.4)	49 (45.4)	
>50	173 (44.6)	59 (54.6)	
Gender			0.831
Female	23 (6.0)	7 (6.5)	
Male	365 (94.0)	101 (93.5)	
HBsAg			0.675
Negative	64 (16.5)	16 (14.8)	
Positive	324 (83.5)	92 (85.2)	
HBVDNA			0.032
≤10^3^	181 (46.6)	63 (58.3)	
> 10^3^	207 (53.4)	45 (41.7)	
Cirrhosis			<0.001
None or mild	190 (49.0)	23 (21.3)	
Moderate or severe	198 (51.0)	85 (78.7)	
Ascites			0.542
Absent or mild	359 (92.5)	98 (90.7)	
Moderate or severe	29 (7.5)	10 (9.3)	
PLT (10E9/L)			0.192
≤100	37 (9.5)	15 (13.9)	
>100	351 (90.5)	93 (86.1)	
ALT (U/L)			0.720
≤50	248 (63.9)	67 (62.0)	
>50	140 (36.1)	41 (38.0)	
AST (U/L)			0.008
≤40	151 (38.9)	27 (25.0)	
>40	237 (61.1)	81 (75.0)	
ALB (g/L)			<0.001
≤40	117 (30.2)	52 (48.1)	
>40	271 (69.8)	56 (51.9)	
TBIL (μmol/L)			<0.001
≤20.5	335 (86.3)	77 (71.3)	
>20.5	53 (13.7)	31 (28.7)	
PT (s)			0.094
≤13.5	323 (83.2)	97 (89.8)	
>13.5	65 (16.8)	11 (10.2)	
AFP (ng/ml)			0.336
≤400	135 (34.8)	43 (39.8)	
>400	253 (65.2)	65 (60.2)	
Child-pugh score			<0.001
5	344 (88.7)	82 (75.9)	
6	39 (10.0)	24 (22.2)	
>6	5 (1.3)	2 (1.9)	
Number of tumor (s)			<0.001
Single	263 (67.8)	35 (32.4)	
Multiple	125 (32.2)	73 (67.6)	
Tumor distribution			<0.001
Uni-lobar	351 (90.5)	53 (49.1)	
Bi-lobar	37 (9.5)	55 (50.9)	
Size of largest nodule (cm)			0.172
<5	56 (14.4)	23 (21.3)	
5–10	214 (55.2)	51 (47.2)	
>10	118 (30.4)	34 (31.5)	
Tumor thrombus			
Vp1	22 (5.7)	1 (0.9)	<0.001
Vp2	111 (28.6)	9 (8.3)	
VP3	237 (61.1)	57 (52.8)	
Vp4	18 (4.6)	41 (38.0)	
Pre-treatment			
None	306 (78.9)	40 (37.0)	
Surgery	7 (1.8)	12 (11.1)	
TACE	54 (13.9)	94 (87.0)	
RFA/PMCT	6 (1.5)	14 (13.0)	
HAIC	15 (3.9)	16 (14.8)	
Follow-up treatment			
Surgery	7 (1.8)	3 (2.8)	
TACE	169 (43.6)	41 (38.0)	
RFA/PMCT	51 (13.1)	12 (11.1)	
TAI	12 (3.1)	15 (13.9)	
Radiotherapy	7 (1.8)	6 (5.6)	
Sorafenib	25 (6.4)	–	

* *No. (%)*.

*PSM, propensity score matching; HBsAg, hepatitis B surface antigen; PLT, blood platelet; ALT, alanine aminotransferase; AST, aspartate aminotransferase; ALB, albumin; TBIL, total bilirubin; PT, prothrombin time; AFP, alpha fetoprotein; TACE, transcatheter arterial chemoembolization; RFA, radiofrequency ablation; PMCT, percutaneous microwave coagulation therapy; HAIC, hepatic artery infusion chemotherapy*.

After a 1:1 PSM, 72 pairs of patients were selected. The basic clinical characteristics between the two groups were almost consistent (Table 2). Initially treated patients still differed, for 50 (69.4%) in the resection-based group and 20 (27.8%) in the sorafenib-based group. As for additional treatments, 4 patients received sorafenib after surgery and 3 patients received surgical resection after sorafenib treatment.

**Table 2:** Baseline clinical characteristics of patients after PSM.

Characteristic^*^	**Therapy**		***P-*value**
	**Resection-based (*n* = 72)**	**Sorafenib-based (*n* = 72)**	
Age (y)			0.238
≤50	27 (37.5)	34 (47.2)	
>50	45 (62.5)	38 (52.8)	
Gender			1.000
Female	5 (6.9)	5 (6.9)	
Male	67 (93.1)	67 (93.1)	
HBsAg			0.061
Negative	19 (26.4)	10 (13.9)	
Positive	53 (73.6)	62 (86.1)	
HBVDNA			0.133
≤10^3^	33 (45.8)	42 (58.3)	
>10^3^	39 (54.2)	30 (41.7)	
Cirrhosis			0.218
None or mild	28 (38.9)	21 (29.2)	
Moderate or severe	44 (61.1)	51 (70.8)	
Ascites			0.275
Absent or mild	70 (97.2)	66 (91.7)	
Moderate or severe	2 (2.8)	6 (8.3)	
PLT (10E9/L)			0.614
≤100	8 (11.1)	10 (13.9)	
>100	64 (88.9)	62 (86.1)	
ALT (U/L)			0.590
≤50	51 (70.8)	48 (66.7)	
>50	21 (29.2)	24 (33.3)	
AST (U/L)			0.230
≤40	31 (43.1)	24 (33.3)	
>40	41 (56.9)	48 (66.7)	
ALB (g/L)			0.053
≤40	19 (26.4)	30 (41.7)	
>40	53 (73.6)	42 (58.3)	
TBIL (μmol/L)			0.533
≤20.5	59 (81.9)	56 (77.8)	
>20.5	13 (18.1)	16 (22.2)	
PT (s)			0.532
≤13.5	68 (94.4)	65 (90.3)	
>13.5	4 (5.6)	7 (9.7)	
AFP (ng/ml)			0.230
≤400	24 (33.3)	31 (43.1)	
>400	48 (66.7)	41 (56.9)	
Child-pugh score			0.386
5	61 (84.7)	57 (79.2)	
>5	11 (15.3)	15 (20.8)	
Number of tumor (s)			0.736
Single	30 (41.7)	32 (44.4)	
Multiple	42 (58.3)	40 (55.6)	
Tumor distribution			0.278
Uni-lobar	53 (73.6)	47 (65.3)	
Bi-lobar	19 (26.4)	25 (34.7)	
Size of largest nodule (cm)			0.974
<5	17 (23.6)	16 (22.2)	
5-10	34 (47.2)	34 (47.2)	
>10	21 (29.2)	22 (30.6)	
Tumor thrombus			0.143
Vp1	2 (2.8)	1 (1.4)	
Vp2	10 (13.9)	9 (12.5)	
VP3	51 (70.8)	42 (58.3)	
VP4	9 (12.5)	20 (27.8)	
Pre-treatment			
None	50 (69.4)	20 (27.8)	
Surgery	0 (0)	12 (16.7)	
TACE	13 (18.1)	42 (58.3)	
RFA/PMCT	2 (2.8)	12 (16.7)	
HAIC	9 (12.5)	11 (15.3)	
Follow-up treatment			
Surgery	2 (2.8)	3 (4.2)	
TACE	27 (37.5)	26 (36.1)	
RFA/PMCT	6 (8.3)	9 (12.5)	
TAI	7 (9.7)	13 (18.1)	
Sorafenib	4 (5.6)	–	

* *No. (%)*.

*PSM, propensity score matching; HBsAg, hepatitis B surface antigen; PLT, blood platelet; ALT, alanine aminotransferase; AST, aspartate aminotransferase; ALB, albumin; TBIL, total bilirubin; PT, prothrombin time; AFP, alpha fetoprotein; TACE, transcatheter arterial chemoembolization; RFA, radiofrequency ablation; PMCT, percutaneous microwave coagulation therapy; HAIC, hepatic artery infusion chemotherapy*.

### Overall Survival Analysis

**Figure 2:**
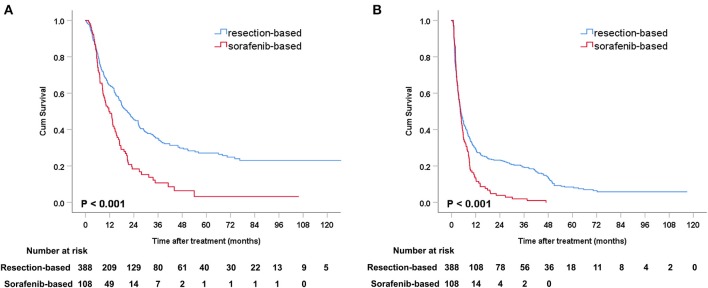
Kaplan-Meier curves of survival outcomes after resection and sorafenib treatment in all patients. **(A)** Overall survival and **(B)** progression-free survival.

Before PSM, the median OS was 20.7 months (95% CI: 16.9–24.5) in the resection-based group and 11.6 months (95% CI: 8.4–14.9) in the sorafenib-based group (*p* < 0.001). The median PFS was 4.7 months (95% CI: 3.8–5.5) in the resection-based group and 4.4 months (95% CI: 3.6–5.2) in the sorafenib-based group (*p* < 0.001). The 6-, 12-, and 24-month OS rates in the resection-based group were 74.0, 55.0, and 33.9%, respectively, and in the sorafenib-based group they were 71.3, 45.4, and 13.0%, respectively. The 6-, 12-, and 24-month PFS rates in the resection-based group were 41.8, 28.4, and 20.5%, respectively, and in the sorafenib-based group they were 33.3, 13.0, and 3.7%, respectively. Survival graphs of the different groups of patients are shown in Figure 2.

### Survival Analysis in the Matching Cohort

**Figure 3:**
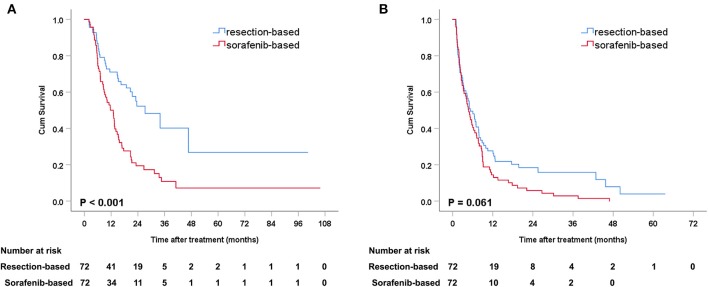
Kaplan-Meier curves of survival outcomes after resection and sorafenib treatment in matched patients. **(A)** Overall survival and **(B)** progression-free survival.

**Figure 4:**
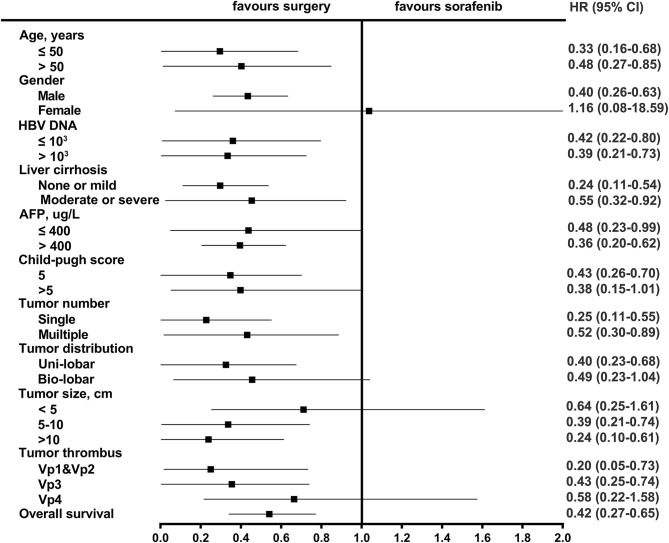
Forest plot for overall survival of the matched cohorts of patients.

After PSM, the median OS was 27.2 months (95% CI: 16.4–38.0) in the resection-based group and 13.0 months (95% CI: 9.6–16.3) in the sorafenib-based group (*p* < 0.001). The median PFS was 5.3 months (95% CI: 3.2–7.4) in the resection-based group and 4.8 months (95% CI: 3.6–6.0) in the sorafenib-based group (*p* = 0.061). The 6-, 12-, and 24-month OS rates in the resection-based group were 80.6, 56.9, and 25.0%, respectively, and in the sorafenib-based group, they were 72.2, 47.2, and 15.3%, respectively. The 6-, 12-, and 24-month PFS rates in the resection-based group were 48.6, 26.4, and 11.1%, respectively, and in the sorafenib-based group, they were 38.9, 13.9, and 5.6%, respectively. Survival graphs are shown in Figure 3. Forest plot analyses of factors associated with OS showed that resection provided a superior clinical benefit in most pre-planned subgroups except in female patients and those with tumor size <5 cm and Vp4 thrombus (Figure 4), as compared to sorafenib. ForPFS, resection only benefited patients with a single tumor (Figure S1).

### Prognostic Factors Analysis of Matched Patients

The risk factors for OS and PFS were analyzed in the matched cohorts (Tables S1, S2). Multivariate analyses identified male (HR = 4.199, 95% CI: 1.023–17.234, *p* = 0.046), patients with ALB > 40 g/L (HR = 0.563, 95% CI: 0.357–0.889, *p* = 0.014), and sorafenib-based treatment (HR = 2.310, 95% CI: 1.481–3.587, *p* < 0.001) as three significant factors associated with OS. Sorafenib-based treatment (HR = 1.391, 95% CI: 0.982–1.968, *p* = 0.063) was a unique factor for RFS in both the univariate and multivariate analysis.

### Progression Analysis of Matched Patients

The position of assessable tumor progression was analyzed in the matched cohorts (Table S3). Patients treated with sorafenib tended to have more intrahepatic progression (91.2 vs. 68.5%, *p* = 0.003). However, there was no statistically significant difference for extrahepatic progression between patients who underwent resection and sorafenib treatment (40.7 vs. 26.3%, *p* = 0.107).

### Survival Analysis of Confounding Factors

Due to the actual treatment, the proportion of initial treated patients in the two groups was inconsistent. Thus, we further explore the prognosis of primary and non-primary patients in the two groups. Patients who underwent non-primary resection had better OS (mOS, 34.0 vs. 18.2 months, *p* = 0.005) but similar PFS (mPFS, 4.1 vs. 4.8 months, *p* = 0.885) as compared to those who received primary resection (Figure S2). No significant survival difference was found between patients received primary and non-primary sorafenib treatment (mOS, 13.6 vs. 10.1 months, *p* = 0.565; mPFS, 5.0 vs. 3.9 months, *p* = 0.407; Figure S3). In the resection-based group, 25 patients were treated with sorafenib after surgical resection and our findings showed that they had no superior OS or PFS (mOS, 24.1 vs. 20.7 months, *p* = 0.900; mPFS, 3.7 vs. 4.7 months, *p* = 0.077) than those who did not take sorafenib during the follow-up treatment (Figure S4).

## Discussion

At present, the optimal therapy for advanced HCC remained uncertain. Although most European guidelines recommend targeted therapy as the first-line therapy, there were still a large number of studies confirming that surgery could bring survival benefit (6–8, 17, 22). Our study proved that in the real world, for some selected patients with good liver function and low tumor burden, surgical resection could have significant benefits of survival and disease control. After PSM, in the two groups of patients with similar baseline levels, surgery was still associated with significant increase in OS.

To our knowledge, there are currently a few studies comparing the efficacy of surgery with sorafenib. Costentin CE reported that OS of patients with HCC and MVI undergoing surgical resection was similar to that treated with sorafenib in a multicenter retrospective study, but the tumor states of patients after matching were not consistent and the result was based on a small sample of patients (46 patients vs. 39 patients) (12). Lee et al. and Wang et al. suggested that surgery offered more survival benefits to advanced HCC patients than other treatments including sorafenib and TACE (8, 23). Kokudo et al. reported their results of surgery in a large cohort of more than 2,000 HCC patients with PVTT (17). The mOS in the resection group was 0.88 years longer than that in the non-resection group (2.45 vs. 1.57 years) in a propensity score-matched cohort. However, sorafenib was not included in the non-surgical treatments.

This study included initially treated and non-initially treated HCC patients with MVI. In our study, there were more initially treated patients who received resection than those who received sorafenib, which was in line with the actual treatment process for HCC patients in the Asia-Pacific region. In the sorafenib-based group, initially or non-initially treatment had no effect on OS and PFS. Several researchers indicated that proper therapies prior to sorafenib led to better survival outcomes for HCC patients (24, 25). It implied that in the treatment strategy of advanced HCC, multiple combinations of locoreginal and systemic modalities treatment could be applied, which may have a beneficial therapeutic impact. For the resection-based group, patients who received non-first-line resection had better prognosis. This might suggest that those patients who could undergo surgery could benefit from preoperative neoadjuvant therapies.

Multivariate analysis showed that indicators of liver function seemed to have a greater impact on prognosis than tumor burden, distribution, and tumor thrombus levels. It was confirmed by other researches that attention should be not only paid to tumor-related conditions, but also to the liver function of patients (26, 27).

The results of subgroup analyses showed that almost all subgroups of patients could have greater overall survival benefits from surgery, except for female patients. This was possibly due to the limited number of identifiable and enrollable cases. Patients with all tumor thrombus levels except Vp4 could benefit from surgery and therefore, systemic therapy would be recommendable for advanced patients with PVTT reaching the main portal vein or HVTT reaching the inferior vena cava. It was confusing that patients with tumor size within 5 centimeters had not a significant OS benefit from surgical resection. Given the relatively small number of patients with small tumors in the analyzed two groups (resection vs. sorafenib, 17 vs. 16), it might accidentally cause statistical bias. The wide range of CI (0.25–1.61) indicated the poor sample representation, which probably could not reveal a true clinical phenomenon. Thus, more cases needed to be accumulated to confirm this part of the issue.

In this study, 25 patients received sorafenib after surgery but their prognosis (mOS, 24.1 months, mPFS, 3.7 months) was not significantly different from those who did not take post-operative sorafenib. Bruix Jordi's phase 3, double-blind, placebo-controlled study showed sorafenib is not an effective intervention in the adjuvant setting for HCC following resection or ablation (28). So, the application of post-operative sorafenib was controversial.

To note, the characteristics showed that patients with a higher tumor burden and more severe liver pathology were given sorafenib-based treatment. This was also reflected by the result that sorafenib-treatment as a negative prognostic factor for this group of patients. So resection shall be given careful evaluation for advanced HCC patients.

This study had some limitations. First, this was a retrospective study and although PSM was applied, there may still be some inevitable selection biases. Second, due to multidisciplinary comprehensive treatment strategy for HCC with MVI, patients included in this study had strong heterogeneity. The final result could not get rid of the influence of confounding factors including previous, concomitant and subsequent treatment after surgery and sorafenib. In addition, after the PSM, the number of cases was relatively small. Findings from this study should be further expanded to multicenter to obtain higher-level medical evidence. Besides, it was worth noting that the analyzed patients were coming from an Asia-Pacific region which was known to have a high incidence of hepatitis B virus (HBV) associated HCCs. Given the high prevalence of HBV associated HCC in Caucasians, Hong Kong Liver Cancer (HKLC) score could be operation-prone due to a better stratification for these patients, where BCLC score might be conservative and unbefitting (29, 30). To better manage the treatment for patients with advanced HCC, especially in Caucasian, HKLC score was expected to applied.

In conclusion, our study indicated that, compared with sorafenib, surgical resection might be associated with better survival benefits in resectable HCC patients with MVI and adequate liver function, and should be considered as an important reliable therapy.

## Data Availability Statement

The datasets generated for this study are available on request to the corresponding author.

## Ethics Statement

The analysis of the patient data was reviewed and approved by the Institutional Review Board and Human Ethics Committee at the Sun Yat-sen University Cancer Center (SYSUCC; Guangzhou, China).

## Author Contributions

JM, WW, and R-PG conceived and designed the experiments. JM, S-HL, Q-XW, J-WZ, L-HL, and Y-HL collected the data. JM, W-PL, and Y-HW analyzed the data. JM wrote the paper. All authors have read and approved the final version of the manuscript.

## Conflict of Interest

The authors declare that the research was conducted in the absence of any commercial or financial relationships that could be construed as a potential conflict of interest.
