# Supplementary material for: Resection vs. Sorafenib for Hepatocellular Carcinoma With Macroscopic Vascular Invasion: A Real World, Propensity Score Matched Analytic Study
Source: Front Oncol. 2020 May 5;10:573. doi: 10.3389/fonc.2020.00573 (PMC7214621; doi:10.3389/fonc.2020.00573)
Supplement: Supplementary file 1 [file Table_1.DOCX]

**Table S1.** Univariate and multivariate analysis of risk factors for overall survival.

| Variables | **Overall survival** | | | | | | |  |
| --- | --- | --- | --- | --- | --- | --- | --- | --- |
|  | **Univariate analysis** | | |  | **Multivariate analysis** | | | |
|  | **HR** | **95% CI** | ***P* value** |  | **HR** | **95% CI** | ***P* value** |  |
| Age > 50 (y) | 0.884 | 0.583-1.339 | 0.559 |  |  |  |  |  |
| Male | 3.972 | 0.976-16.163 | 0.054 |  | 4.199 | 1.023-17.234 | 0.046 |  |
| HBsAg positive | 1.512 | 0.867-2.636 | 0.145 |  |  |  |  |  |
| HBV DNA >10^3^ | 1.098 | 0.727-1.658 | 0.657 |  |  |  |  |  |
| Moderate or severe liver cirrhosis | 1.147 | 0.739-1.781 | 0.540 |  |  |  |  |  |
| Moderate or severe ascites | 1.691 | 0.736-3.883 | 0.216 |  |  |  |  |  |
| PLT > 100 (10^9^/L) | 0.907 | 0.581-1.416 | 0.667 |  |  |  |  |  |
| ALT > 50 (U/L) | 0.890 | 0.574-1.381 | 0.605 |  |  |  |  |  |
| AST > 40 (U/L) | 1.237 | 0.802-1.908 | 0.336 |  |  |  |  |  |
| ALB > 40 (g/L) | 0.539 | 0.354-0.822 | 0.004 |  | 0.563 | 0.357-0.889 | 0.014 |  |
| TBIL >20 (mmol/L) | 0.943 | 0.566-1.569 | 0.820 |  |  |  |  |  |
| PT > 13.5 (sec) | 1.927 | 0.992-3.744 | 0.053 |  | 1.159 | 0.562-2.387 | 0.690 |  |
| AFP > 400 (ng/ml) | 1.159 | 0.758-1.773 | 0.495 |  |  |  |  |  |
| Child-pugh score >5 | 1.552 | 0.949-2.537 | 0.080 |  | 1.101 | 0.630-1/923 | 0.737 |  |
| Multiple tumor | 1.539 | 1.005-2.357 | 0.047 |  | 1.491 | 0.970-2.293 | 0.068 |  |
| Bilateral distribution | 1.313 | 0.850-2.029 | 0.219 |  |  |  |  |  |
| Tumor size >10 (cm) | 1.371 | 0.880-2.134 | 0.163 |  |  |  |  |  |
| Vp4 | 1.308 | 0.820-2.086 | 0.260 |  |  |  |  |  |
| Sorafenib-based | 2.394 | 1.547-3.704 | <0.001 |  | 2.310 | 1.481-3.587 | <0.001 |  |

PLT, platelet count; PT, prothrombin time; ALT, alanine aminotransferase; AST, aspartate aminotransferase; ALB, albumin; TBIL, Total bilirubin; PT, Prothrombin time; VP4, invasion of the main trunk/controlateral branch of the portal vein; AFP, alpha fetoprotein; HR, hazard ratio, CI, Confidence intervals

**Table S2.** Univariate and multivariate analysis of risk factors for progression-free survival.

| Variables | **Progression-free survival** | | | | | | |
| --- | --- | --- | --- | --- | --- | --- | --- |
|  | **Univariate analysis** | | |  | **Multivariate analysis** | | |
|  | **HR** | **95% CI** | ***P* value** |  | **HR** | **95% CI** | ***P* value** |
| Age > 50 (y) | 0.795 | 0.561-1.125 | 0.195 |  |  |  |  |
| Male | 1.557 | 0.759-3.195 | 0.227 |  |  |  |  |
| HBsAg positive | 1.380 | 0.891-2.136 | 0.149 |  |  |  |  |
| HBV DNA >10^3^ | 1.085 | 0.769-1.531 | 0.642 |  |  |  |  |
| Moderate or severe liver cirrhosis | 0.898 | 0.627-1.286 | 0.556 |  |  |  |  |
| Moderate or severe ascites | 1.279 | 0.596-2.745 | 0.528 |  |  |  |  |
| PLT > 100 (10^9^/L) | 0.974 | 0.567-1.672 | 0.924 |  |  |  |  |
| ALT > 50 (U/L) | 0.871 | 0.597-1.270 | 0.474 |  |  |  |  |
| AST > 40 (U/L) | 0.961 | 0.671-1.375 | 0.826 |  |  |  |  |
| ALB > 40 (g/L) | 0.800 | 0.558-1.148 | 0.226 |  |  |  |  |
| TBIL >20 (mmol/L) | 0.742 | 0.473-1.166 | 0.196 |  |  |  |  |
| PT > 13.5 (sec) | 1.496 | 0.803-2.789 | 0.204 |  |  |  |  |
| AFP > 400 (ng/ml) | 1.307 | 0.916-1.865 | 0.140 |  |  |  |  |
| Child-pugh score >5 | 0.772 | 0.484-1.230 | 0.276 |  |  |  |  |
| Multiple tumor | 1.297 | 0.914-1.840 | 0.146 |  |  |  |  |
| Bilateral distribution | 1.013 | 0.696-1.474 | 0.947 |  |  |  |  |
| Tumor size >10 (cm) | 1.180 | 0.813-1.712 | 0.384 |  |  |  |  |
| Vp4 | 1.080 | 0.709-1.644 | 0.719 |  |  |  |  |
| Sorafenib-based | 1.391 | 0.982-1.968 | 0.063 |  | 1.391 | 0.982-1.968 | 0.063 |

PLT, platelet count; PT, prothrombin time; ALT, alanine aminotransferase; AST, aspartate aminotransferase; ALB, albumin; TBIL, Total bilirubin; PT, Prothrombin time; VP4, invasion of the main trunk/controlateral branch of the portal vein; AFP, alpha fetoprotein; HR, hazard ratio, CI, Confidence intervals

**Table S3.** The position of tumor progression analysis

|  | **Resection-based** | **Sorafenib-based** | ***P* value** |
| --- | --- | --- | --- |
| Evaluable patients^*^ | 54 (100) | 57 (100) |  |
| [Intrahepatic](javascript:;) | 37 (68.5) | 52 (91.2) | 0.003 |
| Tumor | 36 (66.7) | 46 (80.7) |  |
| Tumor thrombus | 12 (22.2) | 18 (31.6) |  |
| [Extrahepatic](javascript:;) | 22 (40.7) | 15 (26.3) | 0.107 |
| Lung | 18 (33.3) | 7 (12.3) |  |
| Bone | 1 (1.9) | 3 (5.3) |  |
| Lymph gland | 5 (9.3) | 4 (7.0) |  |
| Adrenal gland | 1 (1.9) | 1 (1.8) |  |
| Other parts | 3 (5.6) | 3 (5.3) |  |

^*^ No. (%)

Figure legends

Figure S1. Forest plot for progression-free survival of matched patients

**Figure S2.** Kaplan-Meier curves of survival outcomes for initial and non-initial resection in the resection-based group. (A) overall survival, (B) progression-free survival

**Figure S3.** Kaplan-Meier curves of survival outcomes for initial and non-initial sorafenib in the sorafenib-based group. (A) overall survival, (B) progression-free survival

Figure S4. Kaplan-Meier curves of survival outcomes for adjuvant and non-adjuvant sorafenib in the resection-based group. (A) overall survival, (B) progression-free survival
