# Supplementary figures and images for: Resection vs. Sorafenib for Hepatocellular Carcinoma With Macroscopic Vascular Invasion: A Real World, Propensity Score Matched Analytic Study
Source: Front Oncol. 2020 May 5;10:573. doi: 10.3389/fonc.2020.00573 (PMC7214621; doi:10.3389/fonc.2020.00573)

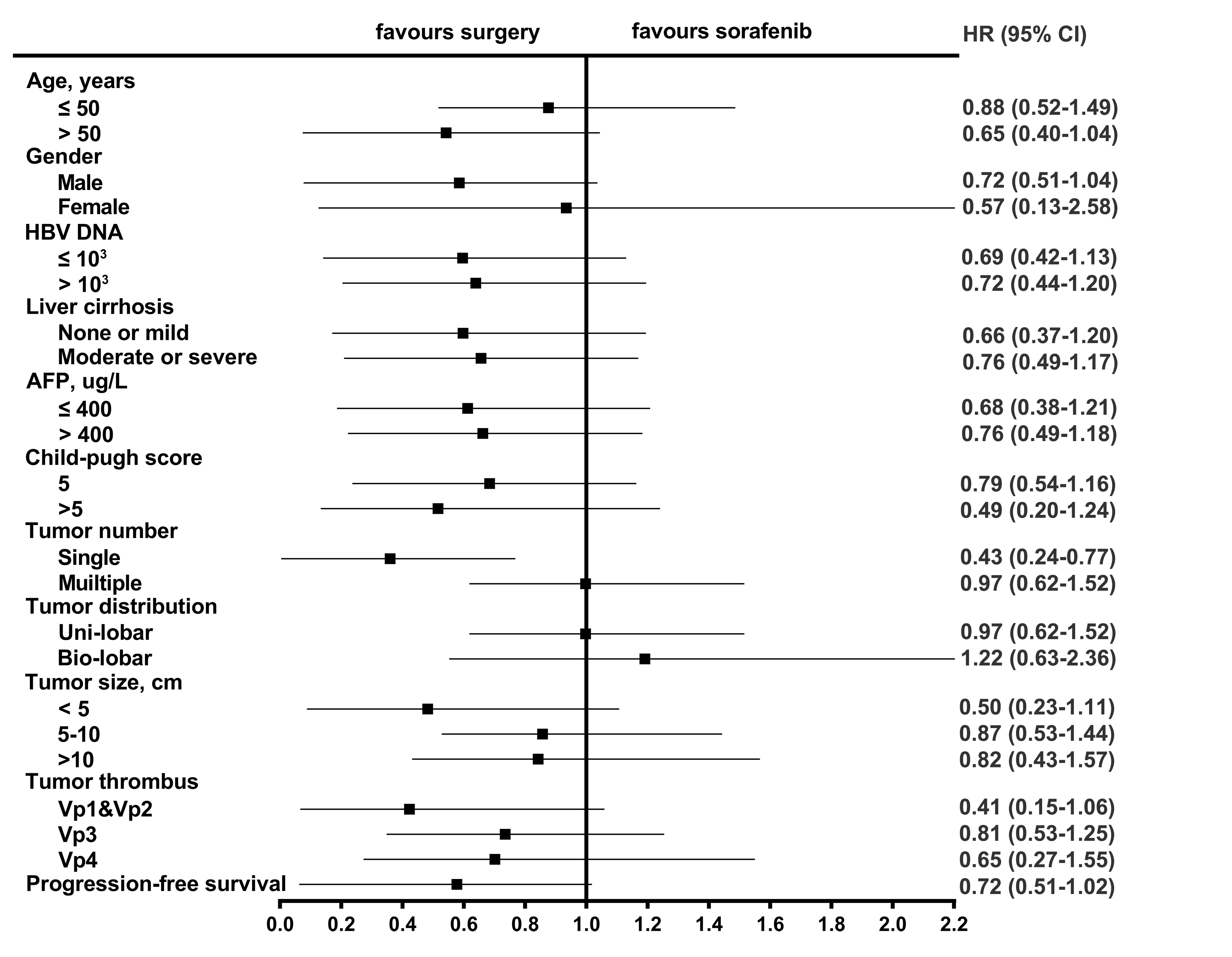

Supplement: Supplementary file 2 [file Image_1.TIF]

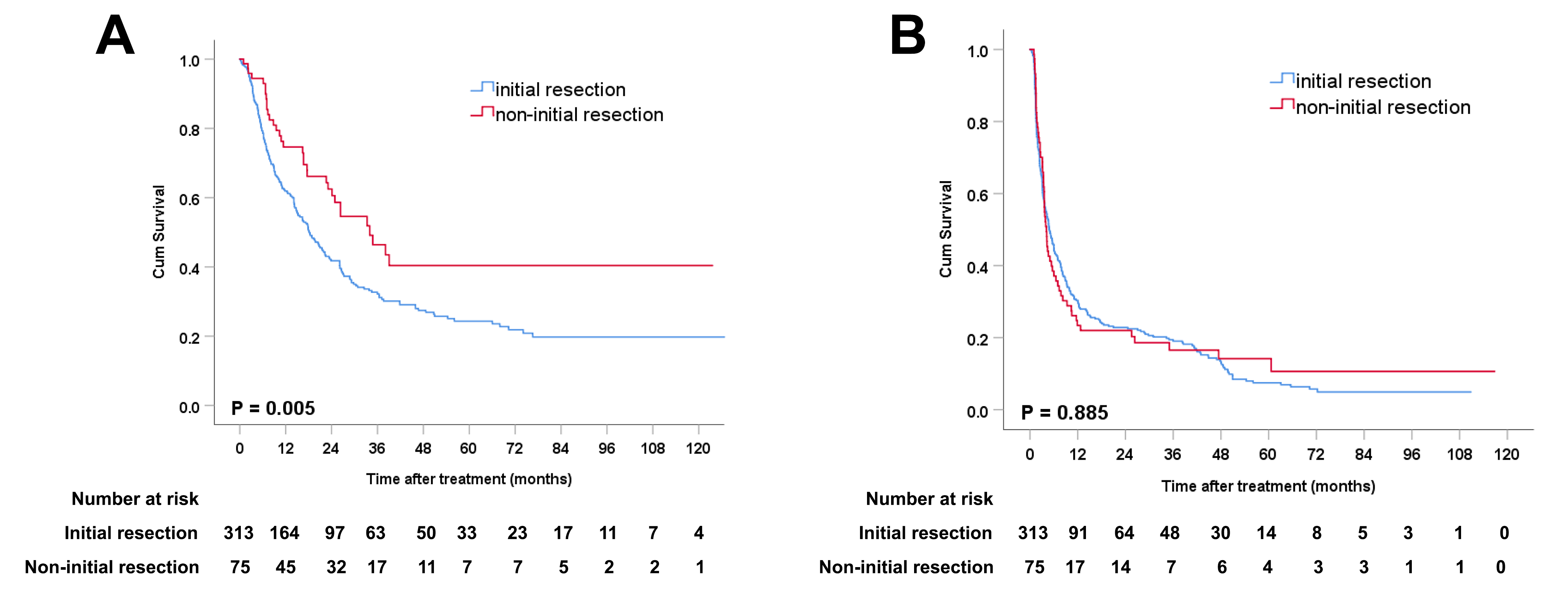

Supplement: Supplementary file 3 [file Image_2.TIF]

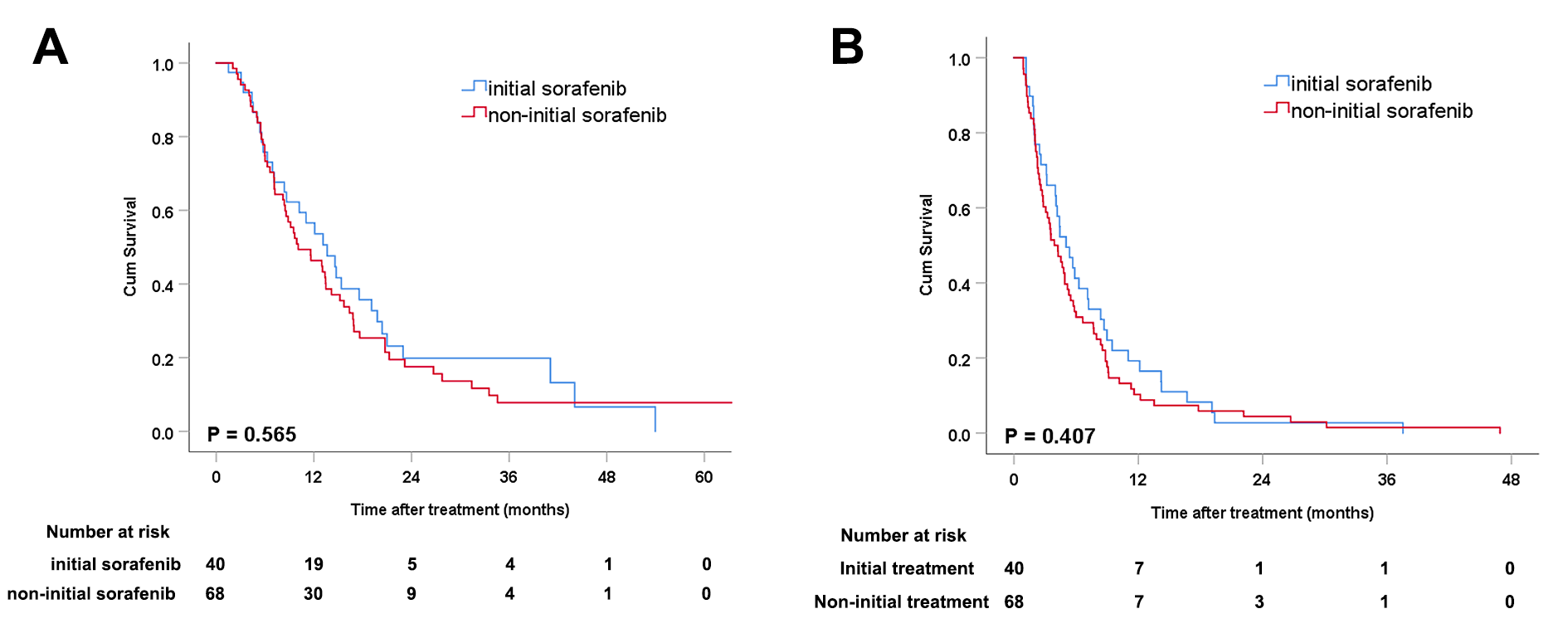

Supplement: Supplementary file 4 [file Image_3.TIF]

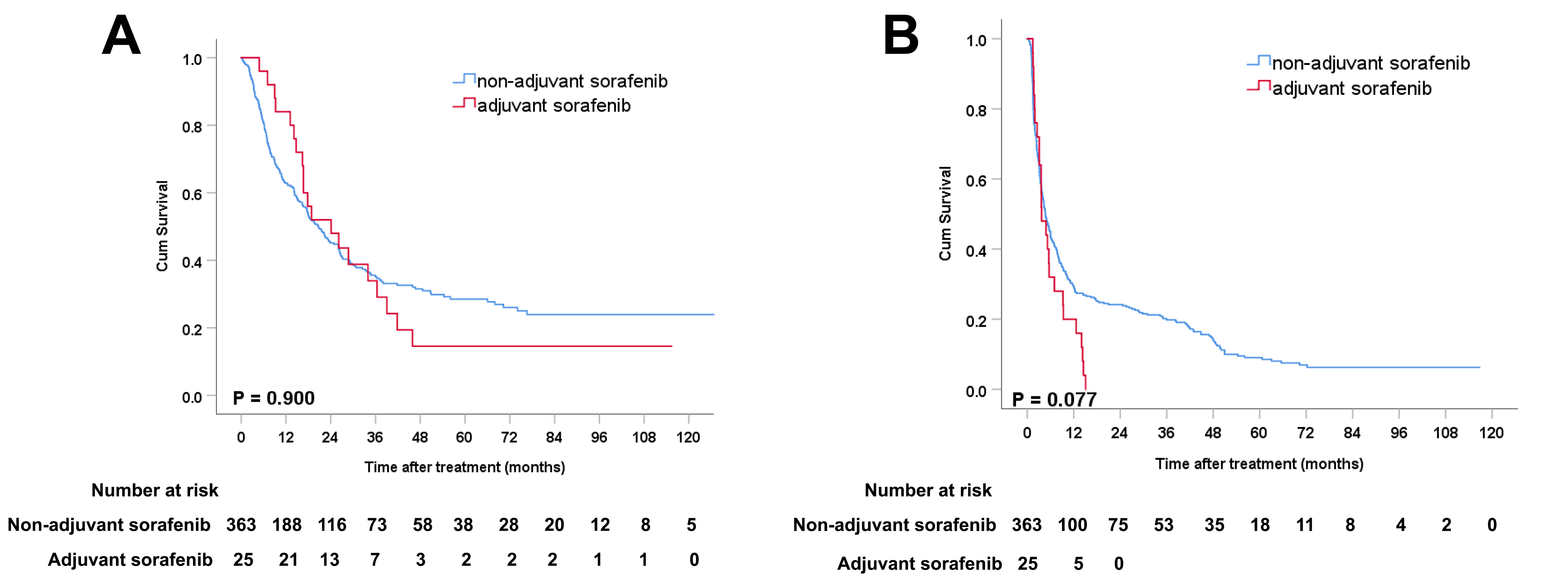

Supplement: Supplementary file 5 [file Image_4.TIF]
